# Presence of apoptotic and nonapoptotic disseminated tumor cells reflects the response to neoadjuvant systemic therapy in breast cancer

**DOI:** 10.1186/bcr1611

**Published:** 2006-10-24

**Authors:** Tanja Fehm, Sven Becker, Graziella Becker-Pergola, Karl Sotlar, Gerhard Gebauer, Silke Dürr-Störzer, Hans Neubauer, Diethelm Wallwiener, Erich-Franz Solomayer

**Affiliations:** 1 Department of Obstetrics and Gynecology, University of Tuebingen, Calwerstrasse 7, D-72076 Tuebingen, Germany; 2 Department of Pathology, University of Tuebingen, Calwerstrasse 7, D-72076 Tuebingen, Germany; 3 Department of Gynecology and Obstetrics, University of Heidelberg, Vossstrasse 9, 69115 Heidelberg, Germany

## Abstract

**Introduction:**

Neoadjuvant systemic therapy (NST) is an established strategy to reduce tumor size in breast cancer patients prior to breast-conserving therapy. The effect of NST on tumor cell dissemination in these patients is not known. The aim of this study was to investigate the incidence of disseminated tumor cells (DTC), including apoptotic DTC, in breast cancer patients after NST, and to investigate the correlation of DTC status with therapy response.

**Methods:**

Bone marrow aspiration was performed in 157 patients after NST. DTC were detected by immunocytochemistry using the A45–B/B3 anticytokeratin antibody. To detect apoptotic DTC the antibody M30 (Roche Diagnostics, Germany) was used, which detects a neo-epitope expressed only after caspase cleavage of cytokeratin 18 during early apoptosis.

**Results:**

The incidence of DTC in breast cancer patients was 53% after completion of NST. Tumor dissemination was observed more frequently in patients with no change/progressive disease (69%) than in patients with partial remission or complete remission of the primary tumor (46%) (*P *< 0.05). Ten out of 24 patients with complete remission, however, were still bone marrow positive. Apoptotic DTC were present in 36 of 157 (23%) breast cancer patients. Apoptotic cells only were detected in 14% of the patients with partial remission or complete remission, but were detected in just 5% of the patients with stable disease. Apoptotic DTC were detectable in none of the patients with tumor progression.

**Conclusion:**

The pathological therapy response in breast cancer patients is reflected by the presence of apoptotic DTC. Patients with complete remission, however, may still have nonapoptotic DTC. These patients may also benefit from secondary adjuvant therapy.

## Introduction

Neoadjuvant systemic therapy (NST) has become the standard treatment for locally advanced breast cancer. The major aim of systemic therapy in these patients is to facilitate breast-conserving surgery. In recent years NST has also been offered to patients with smaller tumors who were expected to receive systemic therapy, since NST offers the possibility for *in vivo *chemosensitivity testing [1-4]. Moreover, prognostic information can be obtained based on the pathologic response to chemotherapy. Patients with complete remission of the primary tumor have a better clinical outcome compared with those with partial remission or compared with nonresponders [5,6].

Local therapy response is normally assessed by palpation and imaging techniques including ultrasound and mammography. The efficacy of neoadjuvant treatment on disseminated tumor cells (DTC) is assumed to be correlated to local therapy response. Complete remission is considered a surrogate marker for complete eradication of micrometastatic disease. However, 13–25% of patients with pathological complete response develop metastatic disease over 5 years [1,5-8].

Eradication of a minimal residual disease may be monitored by serial bone marrow analysis during NST, offering new insights into the effects of systemic therapy on minimal residual disease. DTC can be detected in 30–40% of untreated primary breast cancer patients prior to surgery [9-12]. Tumor cell persistence after removal of the primary tumor and adjuvant chemotherapy has been suggested to indicate chemotherapy resistance and poor clinical outcome [13-15]. The efficacy of neoadjuvant chemotherapy may therefore be indicated by bone marrow negativity or by the presence of disseminated apoptotic cells that were susceptible to cytotoxic agents.

The main focus of this study was to investigate the incidence of DTC, including apoptotic DTC, in patients with breast cancer after primary chemotherapy, and to investigate the correlation of the DTC status with pathological therapy response.

## Patients and methods

### Patients

Primary breast cancer patients (cT1–T4, cN0–N2) who had received systemic therapy and who had undergone surgery at the Department of Gynecology and Obstetrics, University Hospital, Tuebingen, Germany from January 2002 until January 2005 were included in the analysis. Exclusion criteria were a previous history of cancer or a secondary malignancy. Both patients treated with endocrine therapy and patients treated with chemotherapy were included in current trials and received standard treatment. Patients in the endocrine therapy group received either letrozole (*n *= 9) or exemestane (*n *= 6). Patients in the chemotherapy group were treated with either taxane-based (*n *= 110) or anthracycline-based (*n *= 32) chemotherapeutic regimens.

The clinical data of patients are summarized in Table 1. The assessment of clinical response included ultrasound, mammography and physical examination. Clinical complete remission was defined as a complete disappearance of the tumor mass. Partial remission was defined as a reduction of the primary tumor size by 50% or more at the time of surgery. Stable disease was defined as no significant increase or a decrease in tumor size. Progressive disease was defined as the development of new, previously undetected lesions or an increase in the size of a preexisting lesion by 25% after at least two treatment cycles.

Three weeks to 4 weeks after completion of the final cycle of systemic therapy, surgery and bone marrow aspiration were performed. Surgical breast and axillary node resection specimens were evaluated for pathologic tumor response. Patients who had no remaining invasive cancer in the breast and who were lymph node negative were considered to have pathological complete response.

### Bone marrow status and immunohistochemistry

Ten millilitres and 20 ml bone marrow were aspirated intraoperatively from the anterior iliac crest prior to surgery as a routine procedure and were processed within 24 hours. All specimens were obtained after written informed consent from the patient. Tumor cell isolation and detection were performed based on the Consensus Recommendations for Standardized Tumor Cell Detection [16]. Mononuclear cells were obtained by density gradient centrifugation (density 1.077 g/ml, Ficoll; Biochrom, Berlin, Germany) and cytospins were prepared (10^6 ^mononuclear cells/spot) using a cytocentrifuge (Hettich, Tuttlingen, Germany).

For detection of cytokeratin-positive tumor cells, slides were fixed in 4% neutral buffered formalin for 10 minutes and were rinsed in phosphate-buffered saline. Automatic immunostaining was performed on the DAKO autostainer using the monoclonal mouse A45–B/B3 antibody (Micromet, München, Germany) and the DAKO-APAAP detection kit (DakoCytomation, Glostrup, Denmark) according to the manufacturers' instructions. The A45–B/B3 antibody is directed against common cytokeratin epitopes including the cytokeratin heterodimers 8/18 and 8/19.

For each patient, 2 × 10^6 ^cells were analyzed (two slides). Slides were automatically scanned using the ACIS™ imaging system (ChromaVision, Medical Systems Inc., San Juan, Capistrano, CA, USA). Criteria for evaluation of immunostained cells were based on the criteria of the European ISHAGE Working group for standardization of tumor cell detection [17]. MCF-7 was used as a positive control. Leukocytes from healthy volunteers served as the negative control.

Additional slides from each patient were stained using the M30 antibody (Roche Applied Science, Mannheim, Germany) and the APAAP kit detection method as described above. The antibody reacts with a neo-epitope expressed only after caspase cleavage of cytokeratin 18 during early apotosis [18,19]. Identification of apoptotic tumor cells were based on positive M30 staining and cytomorphological criteria as described elsewhere [20,25-27].

### Statistical analysis

The chi-squared test was used to examine the relationship between bone marrow micrometastases and clinicopathological factors. Statistical analysis was performed by SPSS (version 11.5; SPSS, Chigaco, Illinois, USA). *P *< 0.05 was considered statistically significant.

## Results

### Patients and primary tumor data

A total of 157 patients were included in the analysis. Table 1 summarizes the patients' characteristics. The median age of patients was 53 years (range, 24–85 years). Most patients had ypT2–ypT4 tumors after NST. Positive lymph nodes were seen in 52% of patients. The predominant tumor type was the invasive ductal carcinoma (97 of 157 cases). Sixty-two per cent of patients were positive for estrogen receptor and 49% were positive for progesterone receptor. The HER2 status was positive in 25% of patients.

### Response to primary systemic therapy

Response rates to therapy are presented in Table 1. Of the 157 patients, 142 received chemotherapy. The remaining patients were treated with endocrine therapy. Twenty-four out of 157 patients showed pathological complete remission. Partial remission was seen in 84 patients. Stable disease or progression was observed in 42 patients and five patients, respectively.

### Correlation with prognostic factors

**Figure 1:**
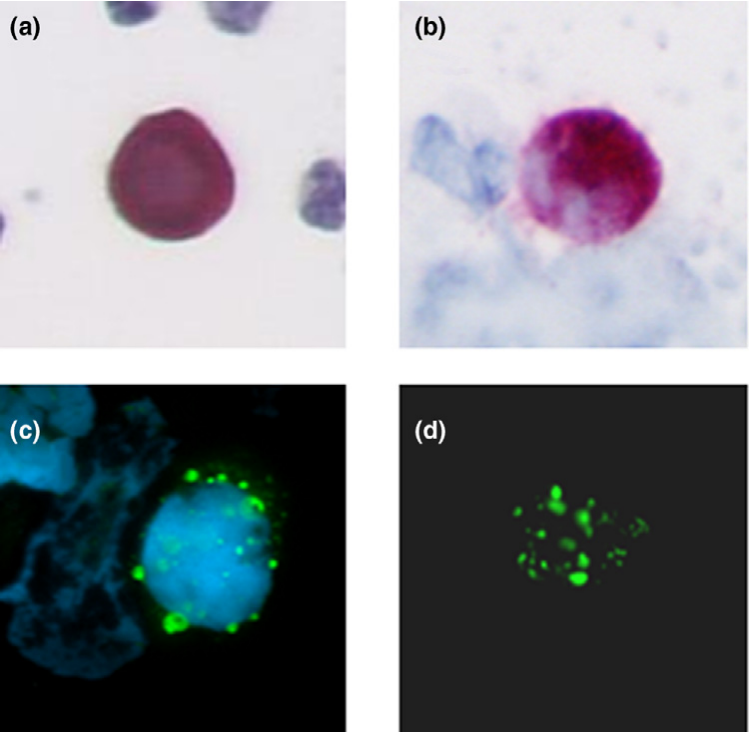
Disseminated apoptotic and nonapoptotic disseminated tumor cells. **(a) **Nonapoptotic disseminated tumor cell (DTC) (detected by the APAAP kit method). **(b) **Apoptotic DTC identified by M30 staining and morphological assessment (for example, membrane blebbing, nuclear shrinkage) (APAAP kit detection method). **(c) **Apoptotic DTC identified by morphological assessment (nuclear shrinkage, cytokeratin inclusions) (DTC detected by a fluorescein-5-isothiocyanate-labeled anticytokeratin antibody). **(d) **Apoptotic cell body without detectable amounts of chromatin.

The incidence of DTC in breast cancer patients was 53% (84 out of 157 patients) after completion of NST (apoptotic DTC were not taken into account for this analysis) (Figure 1a). No correlation could be observed with any of the established prognostic factors, including the lymph node status, the tumor size, the hormone receptor status or the grading. Tumor dissemination was observed more frequently in patients with no change/progressive disease (69%) than in those patients who showed partial remission or complete remission of the primary tumor (46%) (*P *< 0.05). Ten out of 24 (42%) patients with complete remission, however, were still bone marrow positive.

### Presence of apoptotic cells

Bone marrow was analyzed for the presence of apoptotic cells by M30 staining. Apoptotic tumor cells could be detected in 36 out of 157 (23%) breast cancer patients (Figure 1b). Seventeen patients had only apoptotic cells in their bone marrow, whereas 19 patients had both apoptotic cells and nonapoptotic cells. The presence of only apoptotic cells was higher in bone-marrow-positive patients with partial remission or complete remission (14%) compared with those patients with stable disease or progressive disease (4%) (*P *< 0.05). No apoptotic tumor cells were detected in patients with tumor progression (*n *= 5). Interestingly, 10 of the 24 patients with complete remission also had nonapoptotic cells. Data are summarized in Table 2.

## Discussion

Over the past years, neoadjuvant chemotherapy has become increasingly important even in smaller tumors and may to date already be considered a standard strategy in locally advanced disease. Neoadjuvant chemotherapy not only increases the rate of breast-conserving surgery, but also allows an *in vivo *chemosensitivity testing [1]. The response to NST is closely correlated to disease-free survival and overall survival [5,6]. Nevertheless approximately 15–25% of the complete remission patients will develop metastatic disease within 5 years after surgery, suggesting subclinical tumor cell persistence after systemic therapy [5-8]. Since DTC reflect the first step of a subclinical tumor cell spread [20], the aim of the present study was to investigate the bone marrow status in patients after completion of neoadjuvant therapy. Moreover, to further investigate the effect of chemotherapy on residual tumor cells, M30 staining as a marker for apoptosis was applied to DTC and correlated to the clinical and pathological response to therapy of the primary tumor.

### Response of the primary tumor is reflected by bone marrow status

DTC were detected in 53% of patients after completion of neoadjuvant therapy. The highest positivity rate was observed in patients with progressive disease (80%), followed by patients with stable disease (68%) and those with partial remission (48%). Only 10 of 24 (42%) patients with pathological complete remission were DTC positive. The response of the primary tumor was therefore reflected by the presence of DTC. Interestingly, the overall incidence was higher compared with the preoperative positivity rates reported in primary breast cancer patients prior to surgery and not treated by chemotherapy, ranging from 30% to 50% [9-12]. Most of the patients, however, had initially advanced stages with nodal involvement. In general, these patients tend to have higher positivity rates in the bone marrow. In addition, most of the patients still have tumor left despite neoadjuvant chemotherapy. It has been shown that regressing tumors still shed tumor cells into the blood circulation to the same extent as progressing tumors [21]. Therefore, even if patients respond to chemotherapy, cells may be still shed into the bone marrow until removal of the primary tumor. Most of these cells may have no relevance and undergo apoptosis induced by cytotoxic agents.

### Presence of apoptotic cells in bone marrow after systemic treatment

Apoptosis is programmed cell death, which represents a signaling pathway that leads to cellular suicide in an organized manner. Apoptosis is fundamentally different from the necrotic mode of cell death, in which the cells suffer a major insult, resulting in a loss of membrane integrity, swelling and disrupture of the cell. The gold standard for detection of apoptosis has been morphological assessment either with electronic microscopy or with light microscopy [22-28]. Cells are characterized by specific morphologic signs of celllular shrinkage, membrane blebbing (cleavage of cytoskeletal proteins leading to blebbing), nuclear condensation and fragmentation [26]. The alteration of the cytoskeleton takes place first [42] and the intact intermediate filament network is then replaced by cytokeratin inclusions (Figure 1c). The final stages are apoptotic bodies (Figure 1c,d) [28].

Given that apoptosis is the principle mechanism of chemotherapy-induced regression [29], we analyzed the bone marrow aspirates for the presence of apoptotic DTC by immunocytochemical staining with M30 antibody. This antibody reacts with a caspase cleaved epitope of cytokeratin 18 expressed during early apoptosis [18].

No apoptotic DTC could be detected in the bone marrow of patients with progressive disease, whereas the positivity rate of only apoptotic DTC was 4% in patients with stable disease versus 14% in patients with partial remission or complete remission. The presence of only apoptotic DTC was therefore reflected by the therapy response. A subset of patients with stable disease, partial remission or complete remission had both apoptotic cells and nonapoptotic cells, reflecting heterogeneity in terms of therapy responsiveness also observed in the primary tumor [30].

### Clinical relevance of disseminated tumor cells in patients with complete remission

The most interesting group of patients to elucidate the biology of chemoresistance and metastatic disease are those with complete remission of the primary tumor and persisting tumor cells in the bone marrow. The aim of (neo)adjuvant chemotherapy is to eradicate micrometastatic disease to improve disease-free survival and overall survival. Theoretically, if complete remission indicates complete eradication of metastatic disease, no patients with complete remission should have disseminated DTC or should develop metastatic disease during the course of disease. Several studies have shown, in fact, that patients without DTC have the best outcome. However, 13–25% of these patients suffer from systemic relapse. Since no primary tumor is left, one of the sources of metastatic cells is likely to be in the bone marrow. In our study, nonapoptotic DTC could be detected in 10 out of 24 (42%) patients with complete remission. Similar positive rates of tumor cell persistence have also been observed in breast cancer patients after surgery and completion of adjuvant therapy [13-15]. These DTC have the capability to survive in the blood circulation despite cell-matrix loss and are apparently resistant to systemic therapy. Two important steps of the metastatic cascade are therefore fulfilled [21]. Since it has already been demonstrated that patients with persistent DTC have a poor prognosis [13-15], determination of the bone marrow status after neoadjuvant chemotherapy might identify those patients at a high risk for metastatic disease and identify patients who may need secondary therapy.

Clearly not all disseminated cells may necessarily be cells with metastatic potential, however, since the positivity rate of 42% is higher than the expected relapse rate for patients with complete remission, ranging from 13 to 25%. Meng and colleagues [31] have shown that circulating tumor cells can be detected in the blood of breast cancer patients more than 20 years after primary diagnosis without evidence for recurrence, supporting the theory of (lifelong) tumor cell dormancy. An important task will therefore be the identification of those persistent and temporarily dormant cells that will cause a metastatic disease. As a very provocative theory it has been suggested that this subpopulation of persistent DTC may also be seen as cancer stem cells since they are dormant but have the ability to regrow following treatment [32,33]. Identification and further characterization of this subset will offer the chance to understand the mechanism of tumor cell growth and metastatic disease. Furthermore, development of new drugs based on these results will optimize treatment strategies since these disseminated cells are directly linked to metastatic spread.

## Conclusion

The presence of apoptotic tumor cells in bone marrow is reflected by therapy response of the primary tumor to systemic therapy. Patients with complete response, however, still may have nonapoptotic DTC, indicating that the response of the primary tumor does not necessarily reflect the therapeutic effect on DTC. Even patients with complete remission but positive bone marrow status after primary systemic therapy may therefore need secondary adjuvant therapy, which may be based on bisphosphonates or antibody-based strategies, to completely eradicate minimal residual disease.

## Abbreviations

DTC = disseminated tumor cells; NST = neoadjuvant systemic therapy.

## Competing interests

The authors declare that they have no competing interests.

## Authors' contributions

TF, GB-P, SD-S, KS and EFS have made substantial contributions to the conception and design of the study, and to the acquisition, analysis and interpretation of data. TF, GG, SB, DW and HN have been involved in drafting the manuscript or revising it. All authors read and approved the final manuscript.

## Figures and Tables

**Table 1:** Basic characteristics of patients with primary systemic therapy

Characteristic	*n*	Bone marrow positive		*P *value
			
		*n*	%	
Total	157	84	53	-
Menopausal status				0.3
Premenopausal	72	42	58	
Postmenopausal	85	42	49	
Tumor size				0.6
ypT0	24	10	44	
ypT1	61	33	54	
ypT2–ypT4	73	41	56	
Nodal status				0.5
ypN negative	75	38	51	
ypN positive	82	46	56	
Estrogen receptor status				1.0
Negative	39	21	54	
Positive	118	63	53	
Progesterone receptor status				0.7
Negative	68	35	52	
Positive	89	49	55	
HER2 status				
Negative (score 0/+1 by immunohistochemistry)	115	63	54	0.7
Positive (score +2/+3 by immunohistochemistry)	39	20	51	
Grading				0.9
I–II	109	58	53	
III	41	21	51	
Histology				0.2
Ductal	97	48	49	
Lobular	33	17	52	
Others	27	19	70	
Primary systemic therapy				0.3
Chemotherapy	142	78	55	
Hormonal therapy	15	6	40	
Response to neoadjuvant systemic therapy^a^				<0.05^c^
Pathological complete remission	24	10 (4)^b^	42 (17)^b^	
Partial remission^d^	87	42 (11)^b^	48 (13)^b^	
Stable disease	40	27 (2)^b^	68 (5)^b^	
Progressive disease	5	4 (0)^b^	80 (0)^b^	

^a^Not including apoptotic cells (see Table 2). ^b^Number (percentage) of patients with only apoptotic cells. ^c^Complete remission + partial remission (46%) versus stable disease + progressive disease (69%). ^d^Partial remission also includes patients with clinical complete remission but pathological partial remission.

**Table 2:** Bone marrow findings subdivided after response to primary therapy

Bone marrow findings	Poor responder (*n *= 45)			Responder (*n *= 111)		
	
	Progressive disease (*n *= 5)	Stable disease (*n *= 40)	Stable disease + progressive disease (*n *= 45)	Partial remission (*n *= 87)	Complete remission (*n *= 24)	Partial remission + complete remission (*n *= 111)
Disseminated tumor cell negative (*n *= 56)	1 (20%)	11 (28%)	12 (26%)	34 (39%)	10 (42%)	44 (40%)
Only apoptotic cells (*n *= 17)	0 (0%)	2 (5%)	2 (4%)	11 (13%)	4 (17%)	15 (14%)
Nonapoptotic cells + apoptotic cells (*n *= 19)	0 (0%)	7 (18%)	7 (16%)	10 (12%)	2 (8%)	12 (11%)
Nonapoptotic cells (*n *= 64)	4 (80%)	20 (50%)	24 (52%)	32 (37%)	8 (33%)	40 (36%)
